# Vision-Based Measurement of Breathing Deformation in Wind Turbine Blade Fatigue Test

**DOI:** 10.3390/jimaging12040174

**Published:** 2026-04-17

**Authors:** Xianlong Wei, Cailin Li, Zhiyong Wang, Zhao Hai, Jinghua Wang, Leian Zhang

**Affiliations:** Shandong Key Laboratory of Wind Power Equipment Testing, Evaluation and Service Support Technology, Shandong University of Technology, Zibo 255000, China; wangzhy1978@163.com (Z.W.); 23507020864@stumail.sdut.edu.cn (Z.H.); wangjh0102@163.com (J.W.); ziaver@163.com (L.Z.)

**Keywords:** wind turbine blade, fatigue test, vision-based sensing, optical deformation measurement, photogrammetry

## Abstract

Wind turbine blades are subjected to complex environmental conditions during long-term operation, which may lead to structural degradation and performance loss. To ensure structural integrity, fatigue testing prior to deployment is essential. This paper proposes a vision-based method for measuring the full-cycle breathing deformation of wind turbine blades during fatigue testing. The method captures dynamic image sequences of the blade’s hotspot cross-section using industrial cameras and employs a feature-based template matching approach to reconstruct the three-dimensional coordinates of target points. Through coordinate transformation, the deformation trajectories are obtained, enabling quantitative analysis of the blade’s dynamic responses in both flapwise and edgewise directions. A dedicated hardware–software system was developed and validated through full-scale fatigue experiments. Quantitative comparison with strain gage measurements shows that the proposed method achieves mean absolute deviations of 0.84 mm and 0.93 mm in two independent experiments, respectively, with closely matched deformation trends under typical loading conditions. These results demonstrate that the proposed method can reliably capture the global deformation behavior of the blade with millimeter-level accuracy, while significantly reducing instrumentation complexity compared to conventional contact-based approaches. The proposed method provides an effective and practical solution for full-field dynamic deformation measurement in blade fatigue testing, offering strong potential for structural health monitoring and early damage detection in wind turbine systems.

## 1. Introduction

As a kind of clean energy, wind energy has the advantages of large resource reserves and wide distribution, and is suitable for large-scale development, making it an important part of renewable energy. Wind power generation, as the main way of wind energy development and utilization, has huge environmental benefits and potential commercial value, and the scale of the industry is growing worldwide.

Wind turbine blades are the core components of wind turbines that capture wind energy, and they are subjected to complex and variable loads; meanwhile, existing structural design theories are not yet fully mature, so the actual service life of the blades often deviates significantly from the design life. At the same time, the long-term exposure of large-sized blades in the environment, by the wind, rain, snow, sun, and other natural factors of erosion, especially the impact of the wind field, easily leads to cracks, fatigue damage, deformation and other problems within the blade. This not only threatens the structural safety of the blade but also reduces the efficiency of power generation and, in serious cases, may even lead to blade fracture, triggering major economic and safety accidents. Wind turbine blades are subjected to more than a hundred cyclic loads over a service life of about 20 years and experience a variety of extreme operating conditions, resulting in cumulative damage and material property degradation. To evaluate their performance, full-scale structural testing of rotor blades is required [1]. The full-scale structural fatigue test can verify the reasonableness of the blade structural design and the standardization of the manufacturing process, and ensure that the actual performance of the blades put into operation in batch meets the design indexes. At the same time, the optimization of fatigue tests can improve the test efficiency of large wind turbine blades and reduce the test cost, so it has become an important direction of the current theoretical research and technological development.

Early studies of blade breathing deformation were mostly accomplished through finite element simulation and analysis [2]. According to the type of unit used, the finite element method can be divided into two categories: beam model and shell model. The beam model has the advantages of simple modeling and fast calculation by simplifying the blade cross-section characteristics, which is suitable for the global response analysis of the blade and commonly used in the numerical simulation of the design stage [3]; the shell model needs to describe the blade cross-section characteristics in detail, which is complicated to model and less efficient to calculate, but it can not only obtain the blade deformation and load distribution, but also provide detailed information on the stress and strain distribution of the cross-section, which is suitable for the local response analysis, and is usually used as a simulation tool in the calibration stage [4]. Extensive research has been conducted internationally on the calculation methods of test load amplitude for beam and shell models, yielding favorable results [5,6,7,8,9,10,11,12]. However, when the finite element method is used to calculate the load magnitude in biaxial resonant full-scale structural fatigue testing, corresponding calculation methods need to be developed according to the accuracy requirements to improve the efficiency and optimize the subsequent test load distribution. Meanwhile, due to factors such as manufacturing deviations and discrete material properties, it is difficult to fully match the simulation model with the actual model in terms of details. Therefore, some new attempts are needed to test the blade breathing fatigue deformation.

With the continuous development and increasing maturity of sensor technology, the application of various types of sensors in blade fatigue testing has attracted widespread attention and has been thoroughly studied. Traditional contact sensors, such as accelerometers, strain gages, and displacement transducers, are often limited in the number of available measurement positions by the weight of the sensor itself and the impact on the local stiffness of the contact area. In contrast, non-contact sensors (e.g., laser Doppler vibrometers, laser triangulation sensors, and proximity sensors [13]), while not interfering with the dynamics of the structure, typically support only single-point measurements, limiting their applicability to complex tests.

In contrast, non-contact measurement techniques have demonstrated good application in a wide range of measurement fields. In 2020, Luo et al. [14] proposed a novel and low-cost method for static deformation measurement of wind turbine blades, in which the curvature and bending of the blade under different flexural loads can be easily obtained by segmenting the blade model in the laser-scanning point cloud. In 2022, Liao et al. [15] designed an online damage assessment system for fatigue loading tests based on a distributed network bus interconnection mode, which utilized laser sensors to detect and collect blade strain data and established a fatigue damage evaluation system for a 56.5 m blade fatigue test. In 2024, Hu et al. [16] proposed a multi-LiDAR system for measuring large spatial deflections of wind turbine blades, which was based on point cloud modeling. The blade’s 3D coordinates were further extracted using alignment, clustering, and line integration methods, with the measurement error controlled within 3%. However, the analysis of blade fatigue deformation by laser point cloud shows higher accuracy at low amplitude and simple spatial deformation patterns, but is susceptible to noise in the case of large and complex deformations. In 2017, Peyman et al. [17] proposed a multi-camera measurement system based on dynamic spatial data splicing to apply photogrammetry to blade fatigue testing. Park et al. [18] proposed a 3D displacement measurement method using circular target points and three cameras to capture the 3D motion parameters of the structure. In 2018, Xu et al. [19] proposed a multi-target displacement monitoring method based on machine vision technology, which applies to multi-point simultaneous vibration or rotational displacement monitoring of large structures. In 2019, Wu et al. [20] realized the full-field dynamic of the blade using two digital camera displacement parameters and diagnosed blade health through time and frequency domains. Xu et al. [21] summarized the displacement measurement process into three main steps: calibration, target tracking, and displacement calculation, and verified the feasibility and applicability of photogrammetry in multipoint simultaneous vibration measurement of large structures. In 2024, Yang et al. [22] proposed a vision-based biaxial fatigue displacement of wind turbine blade measurement method, which calculates the displacement change by updating the displacement conversion factor in real-time through the ratio of the pixel radius of the target point to the actual radius as a reference. However, most of the above methods use single-view measurement [23], which cannot directly obtain the three-dimensional depth information of the target, and most of the deformation measurements are made outside the blade [24], which cannot take into account the simultaneous deformation of the whole blade cross-section. For this reason, it is of great significance to develop a whole-process 3D dynamic deformation measurement method applicable to the fatigue test of wind turbine blades [25].

Based on existing research, this study proposes a vision-based test method for the whole process of blade-breath deformation by combining the existing machine vision measurement technology. The method employs industrial cameras to acquire dynamic images during blade fatigue tests and realizes accurate measurement of dynamic deformation at hotspot cross-sections through a feature-based template matching algorithm. The measured deformation data provide experimental data support and validation evidence for existing numerical simulations, and fatigue analysis studies reported in the literature [26,27]. The main research work of this paper is as follows: Section 2 systematically presents the vision-based blade breathing deformation measurement methodology, with emphasis on the feature-based template matching technique and the proposed coordinate system conversion framework for blade deformation analysis. Section 3 applies the visual measurement method to displacement analysis of blade root regions during breath fatigue tests, validating its effectiveness and accuracy through two comparative experimental datasets. Section 4 provides an in-depth discussion of the target displacement test results, including error sources and performance limitations. Finally, Section 5 summarizes the research results of this paper and looks forward to possible future improvement directions and research priorities.

## 2. Methods

**Figure 1:**
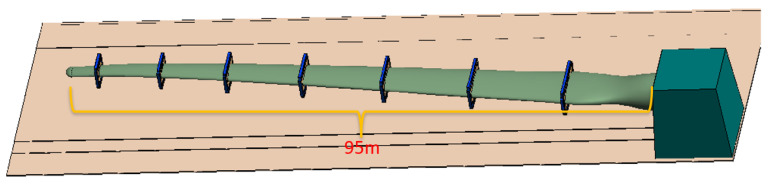
Simulation of wind turbine blade fatigue at the test center.

Full-scale fatigue testing of wind turbine blades is commonly performed on dedicated test benches (Figure 1), with the blade root rigidly fixed and forced excitation applied near the tip to generate large-amplitude flapwise or edgewise oscillations that simulate 20-year service loading. During testing, distinct “breathe” (opening–closing) deformation occurs at internal hot sections and serves as the most direct indicator of web-bonding quality, interlaminar delamination, and accumulated fatigue damage. However, the blade interior forms a completely enclosed, narrow, and dark cavity exceeding 10 m in length, necessitating high-power non-coaxial LED floodlighting for sufficient image brightness. Such strong continuous illumination destroys the retro-reflective principle, causing drastic contrast loss and severe specular highlights on conventional retro-reflective targets. Consequently, recognition rates drop significantly and sub-pixel localisation error deteriorates from the typical ~0.02 pixel to >0.5 pixel [17].

**Figure 2:**
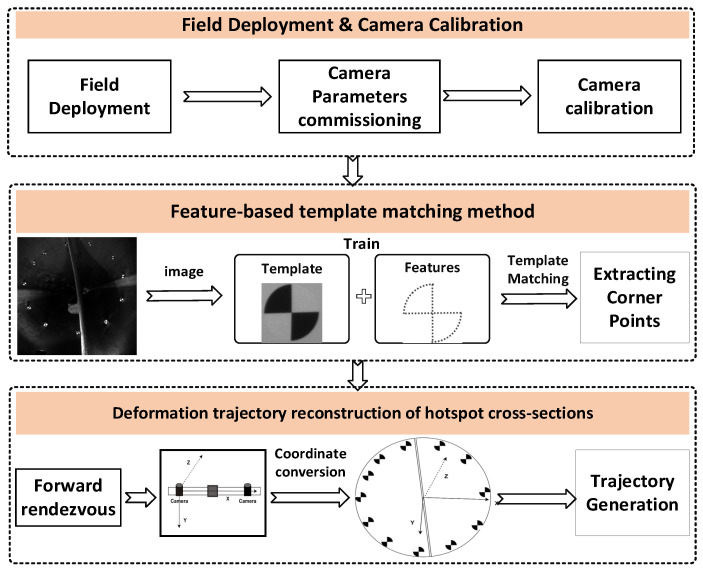
Overall architecture diagram.

To maintain sub-pixel localisation accuracy under this extremely strong non-coaxial illumination condition, conventional retro-reflective coded targets were abandoned in favor of high-precision non-reflective targets (Figure 2). These targets preserve high contrast and sharp edges even under intense ambient light, enabling robust automatic detection and sub-pixel center localisation [27]. The proposed vision-based monitoring method consists of three key stages: field deployment and camera calibration, template matching based on feature points, and Deformation trajectory reconstruction of hotspot cross-sections in wind turbine blades. The complete workflow is illustrated in Figure 2.

### 2.1. Field Deployment and Camera Calibration

**Figure 3:**
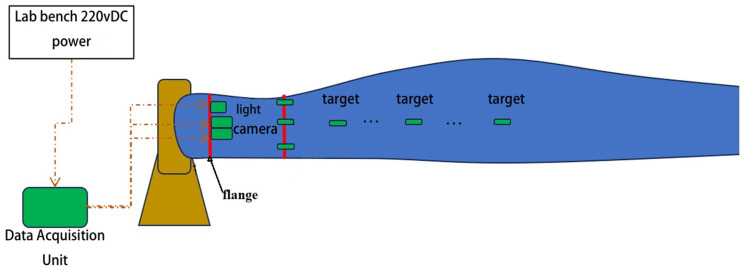
Schematic diagram of device installation.

To ensure the accuracy and reliability of the dynamic deformation measurement system for wind turbine blades, field deployment and camera calibration are essential preparatory steps. First, high-contrast non-reflective targets (100 mm diameter) are uniformly arranged on the blade hot sections, serving as core tracking points for cross-sectional deformation. This layout follows photogrammetric principles, achieving full cross-sectional coverage via multi-point distribution while avoiding the contrast loss of retro-reflective targets under strong LED illumination [17]. To enhance image clarity and internal visibility within the enclosed dark cavity, high-power LED floodlights are installed. This approach effectively mitigates the impact of darkness on target recognition and improves overall image quality. Subsequently, two industrial cameras (as shown in Figure 3) are rigidly mounted inside the blade flange using a stable bracket, ensuring immunity to test-induced vibrations and preventing image jitter from compromising measurement accuracy. The inter-camera baseline is fixed at approximately 0.5 m, with the field of view covering the entire measurement area. Camera parameters are optimized according to experimental requirements, including exposure time, gain, and frame rate (12 fps), to guarantee clear target imaging.

The frame rate of the image acquisition system was set to 12 fps based on both theoretical considerations and practical system constraints. The dominant frequency of the blade breathing deformation during fatigue testing is approximately 0.5 Hz, corresponding to a deformation period of 2 s. According to the Nyquist sampling theorem, the minimum required sampling frequency should be at least twice the signal frequency. In this study, a frame rate of 12 fps provides 24 sampling points per deformation cycle, which is significantly higher than the minimum requirement and ensures accurate reconstruction of the deformation trajectory. In addition to theoretical adequacy, the frame rate selection also considers system stability. Although the industrial camera supports a maximum frame rate of 15 fps at the given resolution, experimental tests showed that operating at 12 fps yields more stable image acquisition, with reduced frame loss and improved synchronization reliability under continuous fatigue loading conditions.

Following deployment, camera calibration is performed. The method is fundamentally close-range photogrammetry, incorporating computer-vision algorithms yet relying primarily on triangulated 3D target coordinates and global bundle adjustment rather than pure natural-feature extraction. A high-precision planar checkerboard (6 cm grid, 0.01 mm alumina flatness) is employed instead of a 3D control field, as the confined blade interior precludes large 3D rig deployment. Multi-pose imaging with a handheld board within the common binocular field of view simplifies operations and reduces costs while achieving over 95% of traditional 3D calibration accuracy [26]. The calibration plane is kept approximately coplanar with subsequent measurement targets, and all poses cover the entire measurement volume.

The calibration procedure is as follows. Checkerboard corners are first used to solve individual camera intrinsics (focal length f, principal point (cx, cy), distortion coefficients k1–k3, p1–p2). With the left-right camera relative pose fixed, the left camera’s intrinsics/extrinsics serve, and extrinsic parameters are used as reference, solving only the right camera’s relative extrinsics to avoid cumulative errors from a world coordinate system [27].

Finally, intrinsic and extrinsic parameters are iteratively refined via Levenberg–Marquardt nonlinear optimization, with uncertainties in interior and relative orientation quantified through covariance matrices and propagated in subsequent bundle adjustment for compensation, ensuring overall calibration accuracy [28].

### 2.2. Feature-Based Template Matching

The feature-based template matching method is used to realize the precise location of the target by feature matching, which is divided into the sample collection and training process, and template matching process.

#### 2.2.1. Sample Collection and Training Process

First, a multi-illumination and multi-scale image acquisition scheme was adopted to construct the training sample set. In a simulated dark environment inside the blade, a total of 800 template images were collected using a high-resolution industrial camera system equipped with an adjustable illumination system.

To ensure sufficient representativeness and reproducibility, the template dataset was explicitly organized according to target instances, illumination conditions, and scale variations. Specifically, in terms of target distribution, templates were collected from targets attached at different angles to account for slight appearance variations caused by installation and surface conditions.

In terms of illumination conditions, for each target, images were acquired under different lighting configurations by varying the illumination angle and intensity, covering typical bright and shadowed conditions inside the blade.

In terms of scale variations, for each illumination condition, templates were collected at multiple distances, resulting in target sizes ranging from 40 × 40 to 200 × 200 pixels, corresponding to near-field and far-field observations.

Through this structured sampling strategy, the 800 templates form a balanced dataset, covering variations in target appearance, illumination, and scale, thereby improving the robustness of the matching process under actual operating conditions. As a result, the template library can be directly reused without the need for repeated reconstruction under similar experimental conditions.

Then for each image, a mask image with the same size as the original image is generated, which is used to mark the effective region of the image matching, but since some images will go beyond the boundary during the image rotation process, it is also necessary to fill the length and width of the mask image, and the specific pixel size depends on the size of the template. Subsequently, based on the given mask image, the feature points are detected, and at the same time, two feature strength thresholds are set to decide whether the feature points are available or not, and the feature points with a strength lower than 30 are regarded as weak features and are not involved in the matching, to filter out the feature points that are not significant enough to reduce the possibility of false matching. The feature point with a strength greater than 60 is regarded as a strong feature, which usually has a high degree of differentiation, can significantly improve the accuracy of matching, and is prioritized for use in the matching process. The corner point strength response value *R* is calculated by the following formula:

(1) $$R=\det\left(M\right)-k\times {\left(trace\left(M\right)\right)}^{2}$$

(2) $$M=\left[\begin{array}{cc} I_{x}^{2} & I_{x}I_{y}\\ I_{x}I_{y} & I_{y}^{2} \end{array}\right]$$

where $I_{x}$ and $I_{y}$ are the gradients of the image in the x and y directions, det and trace denote the determinant and trace of the matrix, respectively, *k* is an empirical constant set to 0.05, and *M* is the autocorrelation matrix. Within the commonly recommended range (0.04–0.06), the value *k* = 0.05 provides an optimal balance between corner localization accuracy and noise suppression, making it suitable for robust feature detection in this study.

Then, the template image is trained to enhance the generalization ability of the template. The feature information extracted above is stored in a category ID. The template images are rotated according to a rotation angle step of 1 degree, and the shape information of each template after the rotation is traversed at the same time; the trained category IDs, shape information, and rotation angle are stored for the subsequent matching process. During the training period, the template image is rotated on the axis of the center point with the formula:

(3) $$T_{in}=\left[\begin{array}{ccc} a & b & \left(1-a\right)\times cx-b\times cy\\ -b & a & b\times cx-\left(1-a\right)\times cy\\ 0 & 0 & 1 \end{array}\right]$$

(4) $$a=scale\times cos\left(angle\right)$$

(5) $$b=scale\times sin\left(angle\right)$$

where a and b are the scale and angle transformation parameters, cx and cy are the positions of the template pixel values, and $T_{in}$ is the transformation matrix.

Finally, since this paper expands the source template image during the training phase, this may affect the matching results by making the matching process access pixels outside the image boundaries. Therefore, during the training phase, the black edges need to be cropped out to restore the original shape of the template.

(6) $$T_{c}=\left[\begin{array}{ccc} 1 & 0 & -tlx\\ 0 & 1 & -tly\\ 0 & 0 & 1 \end{array}\right]$$

(7) $$tlx=templ\left[0\right].tl_{x}$$

(8) $$tly=templ\left[0\right].tl_{y}$$

where $T_{c}$ is the cropping matrix, tlx and tly is the length of the pixels expanded when training the image.

#### 2.2.2. Template Matching Process

The process begins by reading and normalizing the target image, followed by computing NCC scores for each image patch to achieve coarse initial localization [29]. The NCC threshold is determined empirically through validation experiments to balance detection sensitivity and false matches. Lower thresholds increase detection rate but introduce more mismatches, while higher thresholds improve reliability at the cost of missed detections. After repeated testing, the threshold range of 0.80–0.90 was found to be optimal, and a value of 0.85 is adopted for subsequent processing. Fine matching is then performed using template feature points and rotation angles, with iterative optimization to derive the optimal rigid-body transformation matrix (containing rotation and translation) for maximum alignment. Since the template center is invariant to rotation and scaling, the final matching position is determined by tracking the template center:

(9) $$T_{result}=P_{center}\times {\left[\begin{array}{c} cx-tlx+match.x\\ cy-tly+match.y\\ 1 \end{array}\right]}^{-1}$$

where $P_{center}$ is the center of the final training image, $T_{result}$ is the transformation matrix from the original image to the target image, and match.x and match.y are the pixel coordinates on the target image.

Finally, since the template matching process may generate multiple overlapping detection frames, to optimize the detection results, the Non-Maximum Suppression (NMS) algorithm is used to remove redundant frames. Each detection frame is first sorted according to the similarity score, and the overlap between detection boxes is then evaluated starting from the highest-confidence candidate.

The NMS overlap threshold is selected empirically to balance redundancy suppression and target preservation. If the threshold is too low, multiple detections of the same target may remain; if too high, valid detections may be incorrectly removed. In this study, the overlap threshold is set to 0.3, which provides stable performance across different illumination and scale conditions. In this way, each target is detected only once, thereby improving the accuracy and consistency of the final matching results.

### 2.3. Deformation Trajectory Reconstruction of Hotspot Cross-Sections in Wind Turbine Blade

The deformation trajectory of cross-sectional targets is generated through three main steps: corner point extraction, forward intersection, and coordinate system transformation. Corner point extraction identifies strong local features within matched regions, achieving precise localisation of key visual markers and providing a solid foundation for 3D reconstruction. However, mismatches in stereo image pairs may arise due to factors such as illumination variations, image noise, or partial occlusions. To address this, epipolar constraints are imposed to enforce geometric consistency between matched points. By eliminating mismatches that violate epipolar geometry, the accuracy of feature correspondence is significantly improved, thereby enhancing the overall precision and robustness of trajectory reconstruction.

**Figure 4:**
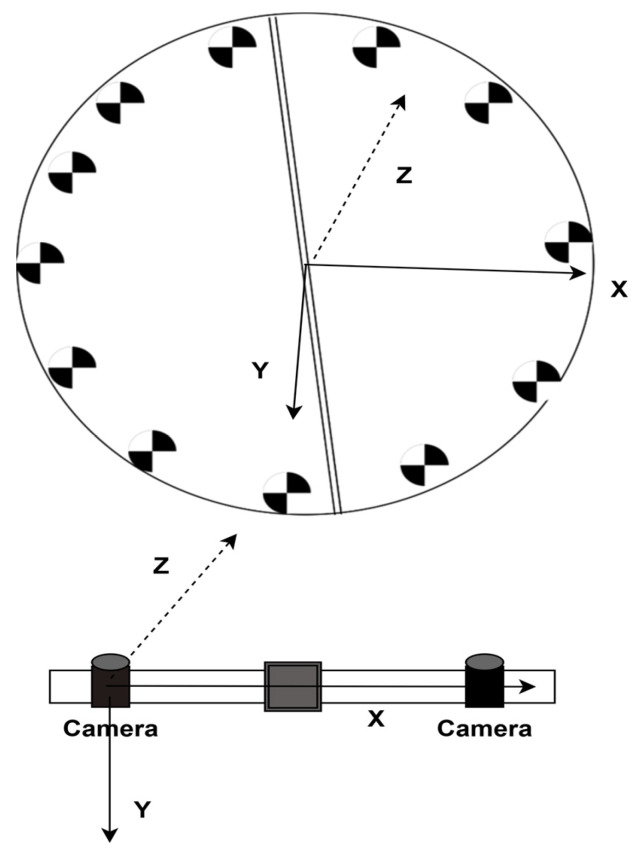
Coordinate system transformation (upper part is the blade cross-section coordinate system, lower part is the blade respirometry coordinate system).

Subsequently, the 3D spatial coordinates of the target point are determined via forward intersection using the cameras. However, these coordinates are expressed in the camera coordinate system (as shown in the lower part of Figure 4), in which the deformation displacement of blade cross-section targets cannot be intuitively visualized. Therefore, a simple transformation is finally applied to convert the camera coordinate system to the blade breathing deformation coordinate system. This system is constructed as follows: the centroid of all targets is computed as the origin; a plane is fitted to the targets, with its normal defining the Z-axis; the stereo camera baseline vector is projected onto the plane to obtain the X-axis; and the Y-axis is determined by the cross product of Z and X. Finally, a rotation matrix R is formed to transform coordinates from the camera system to the blade breathing deformation system [30].

## 3. Experiment

**Figure 5:**
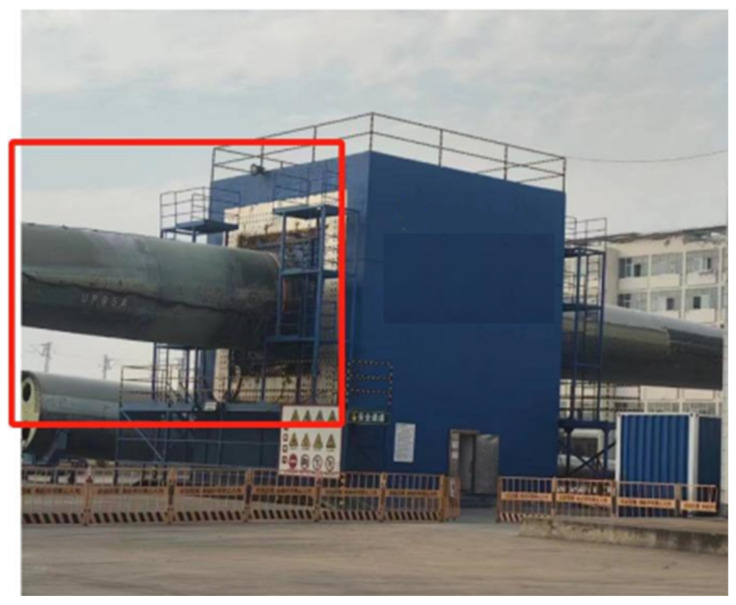
Wind turbine blade at Lianyungang Test Center.

**Figure 6:**
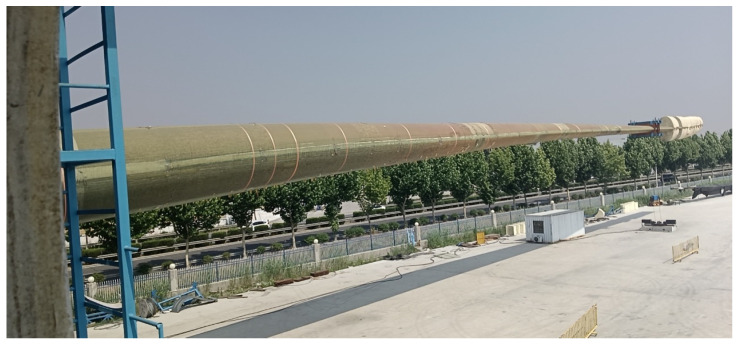
Wind turbine blade at Guannan Test Center.

The experimental program is developed for the internal respiration measurement of two 95 m full-size blades at the Lianyungang (Figure 5) and Guannan test centers (Figure 6), with the blade flange located at the 0.75 m section. The blade flange is located at the 0.75 m section, the blade web start position is 2 m, and the breathing measurement oscillation frequency is about 0.5 Hz. The experiments test the breath deformation of different sections, i.e., the deformation response of the selected discrete measurement point locations on the cross-section in the x, y, and z directions under a certain coordinate system in the operation of the blades.

For image acquisition, a Daheng Imaging MER2-series industrial camera (26-megapixel resolution) (Beijing, China) equipped with a 12 mm fixed-focus lens was employed. The camera was operated at a frame rate of 12 fps to capture the dynamic deformation process. This configuration provides sufficient spatial resolution and imaging stability for accurate feature extraction and three-dimensional reconstruction.

### 3.1. Device Design

The test system is mainly composed of two parts: the hardware device and the software system. The detailed configuration list of the system is shown in Table 1.

**Figure 7:**
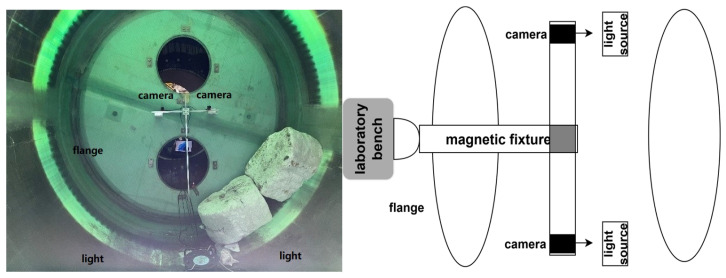
Diagram of the internal mechanism of the flange.

As shown in Figure 7, the data acquisition unit is mounted on the outside of the root of the test bench and connected to the 220 V power supply through the test bench. Two cameras were mounted at the center of the inside of the leaf root flange to synchronize the acquisition of target image data, respectively. The cameras were initially fixed to the flange section, but with the start of the blade fatigue test, it was found that the flange would be displaced with the vibration of the blade, which in turn would affect the accuracy of the data. After several positional tests, it was found that only the experimental bench itself would not shake with the blade, so the final connection was made as shown in Figure 7, where the camera was attached to the experimental bench using a magnetic fixture to minimize the transfer of vibration. The camera baseline is fixed at approximately 0.5 m, with a measurement height (average distance from cameras to blade cross-sections) of about 2.5 m, yielding a height-to-baseline ratio (H/B) of approximately 5.0. This ratio falls within the recommended range for photogrammetry (3–8), ensuring sufficient triangulation accuracy in the depth direction while accommodating the spatial constraints inside the blade.

### 3.2. Camera Calibration

**Figure 8:**
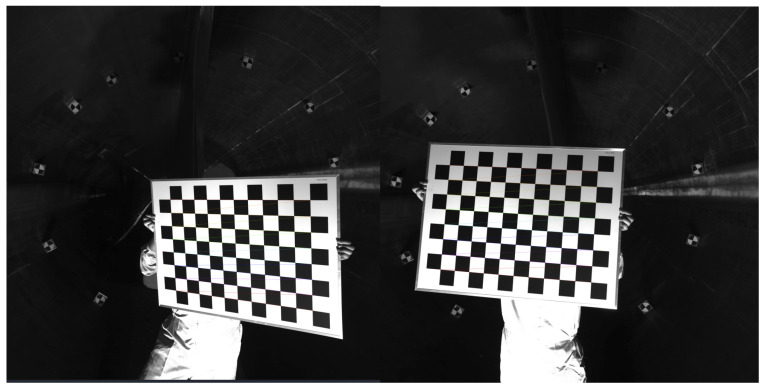
Camera calibration.

Firstly, the target mark is posted on the surface of the wind turbine blade, and the camera parameters are adjusted; then the camera is fixed, and the supplementary light is used to supplement the light source in the dark environment inside the wind turbine blade. In the process of camera calibration, the relative position and attitude of the left and right cameras are fixed, and it is assumed that the internal and external parameters of the left camera are known here, and the camera is calibrated with it as the reference so that the error comes from the relationship between the two only, and there is no need to accumulate the calibration error of the left camera and the world coordinate system, thus improving the calibration accuracy. The image-square coordinates are determined by the pixel coordinates in the image, and the object-square coordinates use the fixed side length (6 cm) of the checkerboard grid as the actual reference coordinates. An aluminum oxide panel with an accuracy of 0.01 mm was chosen for the calibration board (Figure 8), and two 26-megapixel industrial cameras were used. The calibration method used the camera calibration function in the OpenCV [27], and the parameters were adjusted appropriately according to the specific program requirements.

### 3.3. Stereo Image Data Synchronization Acquisition and Processing

**Figure 9:**
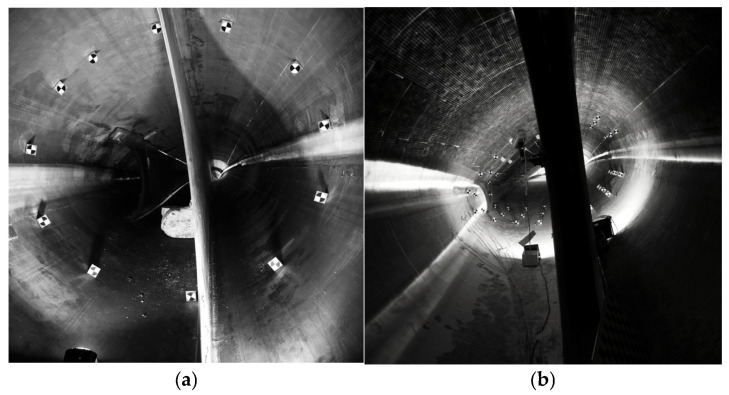
(**a**) Experiment 1 images of a 4 m cross-section from the root of the blade (cross-section at 4 m from root, baseline 50 cm, H/B ≈ 7.0); (**b**) Experiment 2 images of 9.5 m, 10.5 m, and 11.5 m cross-sections from the root of the blade (cross-sections at 9.5 m, 10.5 m, and 11.5 m from root, baseline 42 cm, H/B ≈ 23.8).

The acquisition frame rate of the camera is 12 frames per second, the pixels are 26 million, the exposure time is 30 ms, and the soft trigger mode is used to control the camera for synchronized data acquisition. As shown in Figure 9, when the blade fatigue test load reaches full load, the blade respiration measurement system sends out a trigger command to start synchronized image acquisition, realizing continuous, high frame rate image data acquisition. Although the soft trigger mode may introduce a synchronization error of approximately 10 ms, this temporal offset accounts for only a small fraction of the deformation cycle. Based on a sinusoidal deformation model, the maximum displacement error caused by the time delay can be estimated from the local velocity of the motion. Given that the maximum deformation amplitude is on the order of several centimeters, the corresponding maximum velocity remains relatively low. Under these conditions, a 10 ms time offset results in a displacement error on the sub-millimeter scale, which is significantly smaller than the overall measurement accuracy. The software acquisition system in this paper is the result of secondary development based on the DAHENG camera development kit.

To examine the structural shape changes at different locations during the blade testing process, Experiment 1 was conducted on a cross-section located 4 m from the blade root. A laser-assisted circle-drawing method was used to uniformly arrange 12 targets, enabling a clear and complete analysis of the deformation of the blade surface.

To further verify the robustness of the proposed method in engineering applications, Experiment 2 arranged targets on three cross-sections located 9.5 m, 10.5 m, and 11.5 m from the blade root to comprehensively evaluate the dynamic deformation characteristics at different locations. However, due to occlusion caused by the internal web structure of the blade, some targets could not be simultaneously observed by both cameras, resulting in only 10 valid targets being available for reconstruction. Nevertheless, the spatial distribution of the remaining targets still provides sufficient coverage of the cross-section, particularly in regions with significant deformation, ensuring reliable characterization of the overall breathing deformation behavior.

**Figure 10:**
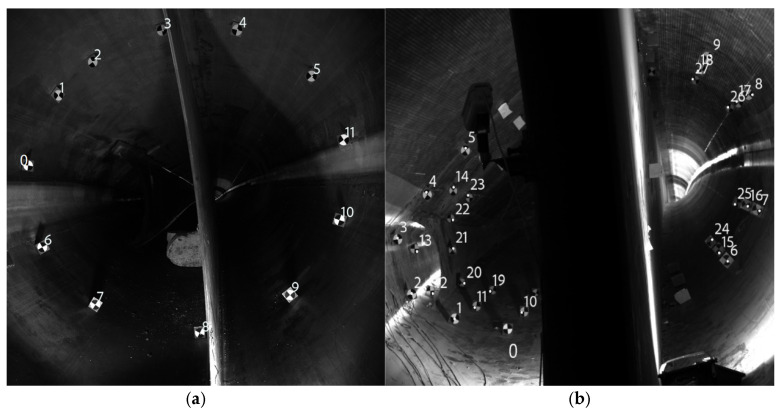
(**a**) Experiment 1: Target numbering diagram for a cross-section of 4 m from the root of the blade. (**b**) Experiment 2: Plots of target numbering for 9.5 m, 10.5 m, and 11.5 m sections from the root of the blade.

**Figure 11:**
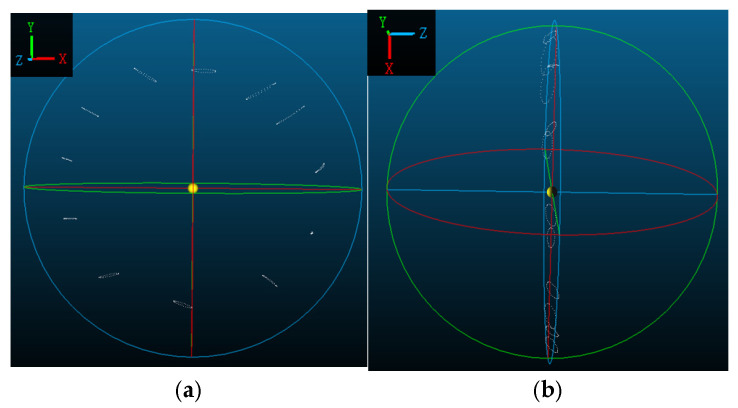
(**a**) Experiment 1: Front view of the trajectory of the cross-section 4 m from the root of the blade. (**b**) Experiment 1: Side view of the trajectory of the cross-section 4 m from the root of the blade.

**Figure 12:**
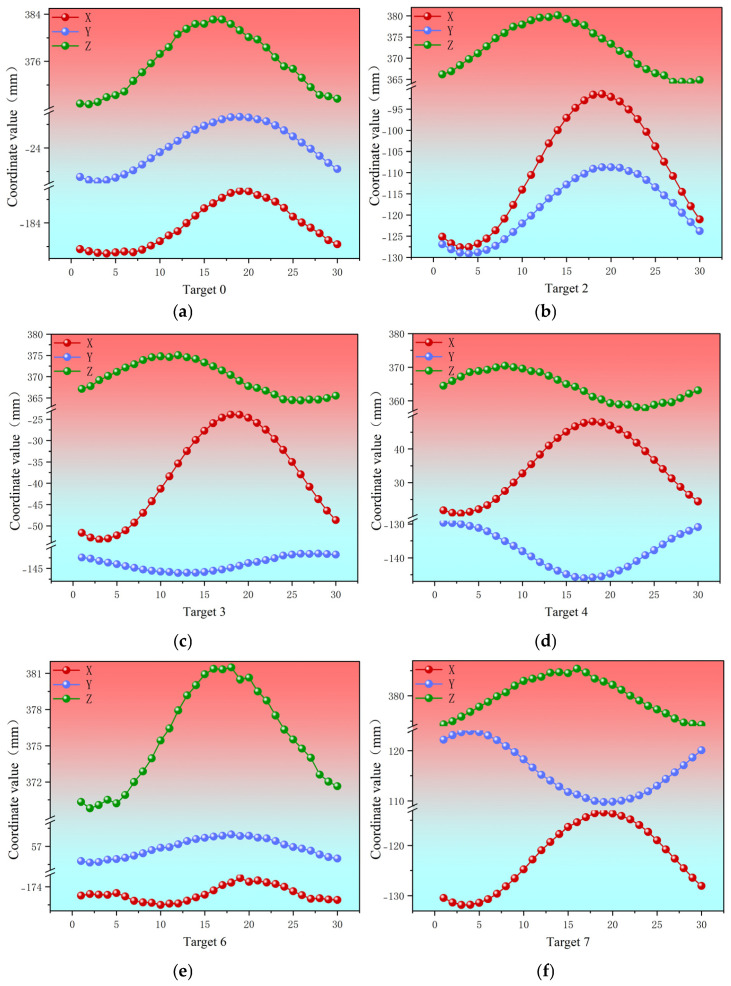
(**a**–**f**) Single-cycle displacement trajectories of the six targets exhibiting the largest deformation amplitudes on the cross-section 4 m from the blade root in Experiment 1. The horizontal axis represents the image sequence number, and the vertical axis shows the coordinate values in the X, Y, and Z directions (unit: mm).

In order to facilitate the subsequent analysis, the target locations are numbered as shown in Figure 10. After a series of processing, such as recognition matching with our method on the acquired images, the cross-section deformation trajectories are obtained as shown in Figure 11 and Figure 12.

## 4. Analysis of Test Results

This chapter presents the performance of the vision system in full-scale biaxial fatigue testing of a wind turbine blade: calibration and system accuracy are reported first, followed by complete 3D breathing deformation trajectories and full-field displacement maps of multiple hot sections, providing reliable data for internal blade deformation studies and test standard improvement.

### 4.1. Calibration Results Analysis

In Experiment 1, the reprojection errors for the left and right cameras are 0.22 pixels and 0.26 pixels, respectively, with a baseline of 48 cm; in Experiment 2, the corresponding errors are 0.36 pixels and 0.41 pixels, with a baseline of 42 cm. In addition to conventional reprojection errors, this study employs the multi-depth planar target validation method to assess system accuracy [31], systematically evaluating precision distribution and stability across the entire measurement volume.

The validation procedure is as follows: checkerboard corners are detected and stereo-matched in calibration images at various depths and poses, and their 3D coordinates are reconstructed via forward intersection. System accuracy is quantified in two aspects: (1) the Euclidean distances between reconstructed corners and their theoretical positions are calculated to obtain the mean point position error, reflecting absolute localisation accuracy; (2) plane fitting is performed on the reconstructed point cloud for each pose, and the standard deviation of distances from points to the fitted plane (plane fitting standard deviation) is computed to assess local flatness and measurement noise.

Results show excellent accuracy in Experiment 1, with a mean point position error of 0.03 cm and a plane fitting standard deviation of 0.02 cm. Equivalent validation in Experiment 2 yields a mean point position error of 0.06 cm and a plane fitting standard deviation of 0.07 cm. All pose results are summarized in Table 2 and Table 3, which systematically present the distribution of point position errors and plane fitting deviations at different depths and poses. Overall, the system exhibits good and consistent measurement performance within the specified working range, confirming the validity of the calibration results and reliability for practical applications.

It should be noted that the stereo configuration in Experiment 2 results in a relatively large height-to-baseline (H/B) ratio, which may theoretically degrade depth (Z-direction) accuracy in photogrammetric reconstruction. According to stereo vision geometry, depth estimation error increases approximately proportionally with the square of the object distance and inversely with the baseline length.

Despite this unfavorable configuration, the experimental results demonstrate that the system maintains acceptable accuracy. As shown in Table 2 and Table 3, the Z-direction errors in Experiment 2 are indeed larger than those in Experiment 1, increasing from approximately 0.027 cm to 0.055 cm on average. This change is consistent with the theoretical inverse relationship between baseline length and depth accuracy. Specifically, the baseline decreases from 48 cm to 42 cm (approximately 12.5% reduction), while the Z-direction error increases by approximately 100%. This nonlinear amplification can be attributed to the combined effects of reduced triangulation geometry and increased sensitivity to image noise at larger object distances, which is consistent with stereo vision theory. Nevertheless, the overall point position error remains within 0.06 cm, satisfying the requirements of millimeter-level deformation measurement.

This robustness can be attributed to several factors: (1) high-resolution imaging (26 MP) improves feature localization accuracy; (2) precise calibration using a high-accuracy checkerboard (0.01 mm) reduces systematic errors; and (3) the use of multi-depth validation ensures stable performance across the measurement volume. Therefore, although the large H/B ratio introduces increased depth uncertainty, the system still achieves reliable measurement performance within the practical working range.

### 4.2. Analysis of Breathing Deformation Results

(1) Experiment 1

Experiment 1 (cross-section at 4 m from the root) captured 200 frames over approximately 16.7 s (equivalent to ~8.3 full fatigue cycles). From both front and side views (Figure 11), the target deformation trajectories appear as smooth ellipses without noticeable irregularities, reflecting the high structural stiffness and stability near the root.

The deformation trajectories of the single-cycle target in Experiment 1 are first analyzed specifically. From the analysis of Figure 11 and Figure 12, it can be seen that the trajectory of the target in the 4 m cross-section is similar to an ellipse, with a larger displacement near the top of the blade, followed by the root of the blade, and the smallest in the middle part. The peak value of the overall deformation of each measurement point on the cross-section is 31.6 mm, and the smallest overall deformation is 10.4 mm, and the targets with larger breath deformations are target No. 2 and target No. 4, which are more than 30 mm. Targets No. 2 and No. 4 exhibit larger breathing deformations, both exceeding 30 mm. Among the 12 targets on the cross-section, the largest total displacement in the X-direction is 35.1 mm at Target No. 2, followed by 29.4 mm at Target No. 3. The maximum total displacement in the Y-direction is 15.5 mm at Target No. 2, with Target No. 7 ranking second at 15.1 mm. In the Z-direction, the peak-to-peak displacement reaches 14.2 mm at Target No. 0, with Target No. 6 following at 11.6 mm.

(2) Experiment 2

**Figure 13:**
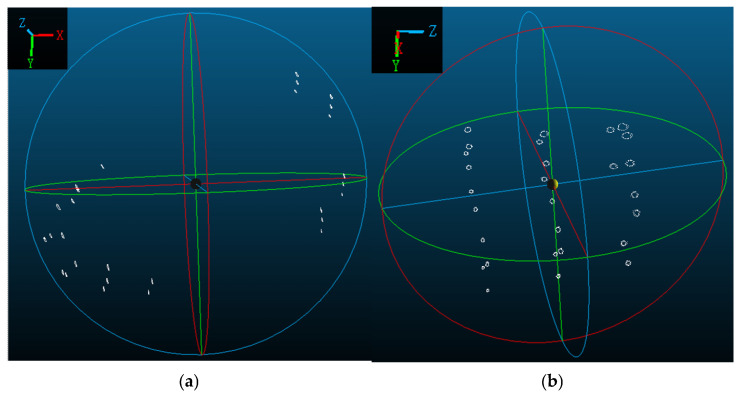
(**a**) Experiment 2 front view of cross-section trajectories at 9.5, 10.5, and 11.5 m from the root of the blade. (**b**) Experiment 2 side view of cross-section trajectories at 9.5, 10.5, and 11.5 m from the root of the blade.

Experiment 2 (cross-sections at 9.5 m, 10.5 m, and 11.5 m from the root) captured 460 frames over approximately 40 s (equivalent to ~16.7 full fatigue cycles). As shown in Figure 13, the trajectories remain generally elliptic but exhibit increased complexity, with slight jitter and irregular fluctuations. This is primarily attributed to the non-rigid characteristics and local aero-structural coupling at the blade’s distal regions, highlighting the progressive reduction in structural stiffness along the span.

**Figure 14:**
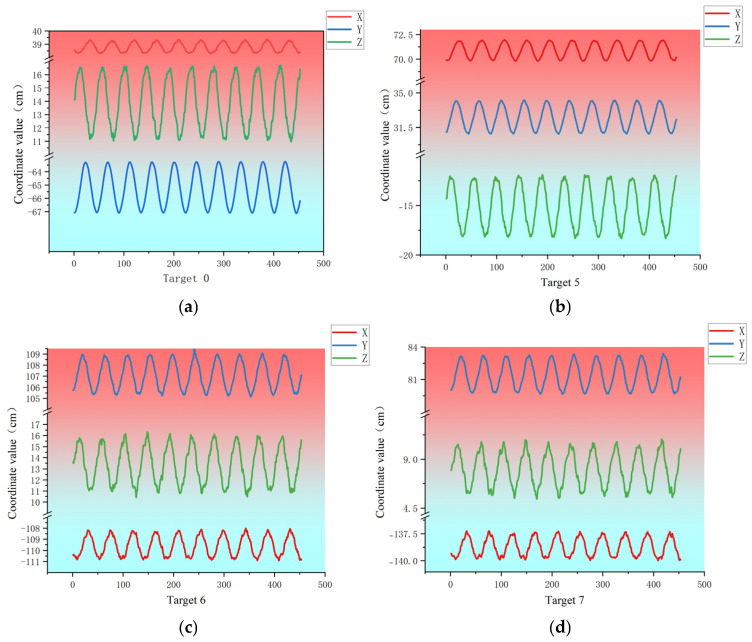
(**a**–**d**) Multi-cycle displacement trajectories of the four targets with the largest deformation amplitudes at the 9.5 m cross-section from the blade root in Experiment 2. The horizontal axis represents the image sequence number, and the vertical axis shows the coordinate values in the X, Y, and Z directions (unit: mm).

In Experiment 1, we only analyzed the deformation displacement of a single cycle, and Experiment 2 will further analyze the deformation displacement of multiple cycles of the blade. As can be seen from Figure 13 and Figure 14, the displacement of the target mark on the blade section of 9.5 m is the smallest, and the displacement at the target mark on the blade section of 11.5 m is the largest. Taking the position of the No. 1 target mark as an example, the maximum displacements of the three sections are 4.8 cm, 7.0 cm, and 9.3 cm, respectively, which shows that the closer the displacement is to the root of the blade, the smaller the displacement is. In a cross-section, the displacement of the middle part is the smallest, the displacement of the upper and lower parts is larger, and the one near the upper part is the largest; the maximum displacements of the three cross-sections are 7.8 cm, 10.7 cm, and 12.01 cm, respectively. Only the deformation data of the 9.5 m cross-section is specifically analyzed here. Due to the obstruction of the field of view, the targets at the upper and lower positions of the cross-section could not be observed by the 2 cameras at the same time, and only 10 target points could be observed. The maximum overall displacement (peak-to-peak value) in the X direction for the 10 targets in the cross-section is 25.1 mm for target No. 6, followed by 20.7 mm for target No. 5, and the maximum overall displacement (peak-to-peak value) in the Y direction is 48.0 mm for target No. 6, followed by 38.4 mm for target No. 0. The maximum overall displacement (peak-to-peak value) in the Z direction is 59.7 mm for target No. 0, followed by 31.3 mm for target No. 7. Due to the short camera baseline (42 cm), the height-to-baseline ratio (H/B) reaches 23.8, Although this short baseline accommodates spatial constraints inside the blade, it inevitably amplifies depth-direction noise and error, manifesting as slight jitter and irregular fluctuations in the trajectories. Nevertheless, the upper and lower positions with large deformation variables can be observed continuously to determine the deformation range of the position when the blade fracture occurs, to improve the subsequent observation program.

### 4.3. Accuracy Verification of the Vision System Using Strain Gages as Reference

As shown in Table 4, under typical fatigue loading conditions, the peak deformation values obtained by the vision-based measurement system exhibit a high degree of consistency with those recorded by strain gages. In this study, foil strain gages (model: BX120-3AA, manufactured in Huangyan, Zhejiang, China) were employed, with a sampling frequency of 50 Hz. The bonding positions of the strain gages were aligned as closely as possible with the corresponding visual targets, with spatial deviations controlled within 5 cm to ensure comparability between the two measurement systems.

The installation process followed standard surface preparation and adhesive curing procedures to ensure reliable strain transfer and measurement stability. Although slight positional discrepancies exist due to practical installation constraints, the measurement locations remain sufficiently close to allow meaningful comparison.

Quantitatively, the overall mean absolute deviation between the two methods is 0.84 mm for Experiment 1 and 0.93 mm for Experiment 2. Both the deformation magnitude and variation trends show strong agreement. This consistency indicates that, despite differences in measurement principles and data acquisition mechanisms, the proposed vision-based approach can accurately capture the global deformation response of the blade during loading, demonstrating high measurement accuracy and robustness.

It is worth noting that certain discrepancies occur in regions exhibiting severe deformation or complex geometric changes, such as the lateral sides of cross-sections or areas with large curvature variations. Further analysis suggests that these local differences primarily arise from the fundamental distinctions in measurement principles: Strain gages measure the average strain over the bonded area, reflecting a spatially integrated response; in contrast, the vision system tracks discrete points in a sequence of images to extract localized displacement or strain data, which better represents the point-wise deformation state. Therefore, in regions with highly non-uniform structural responses, a certain level of systematic deviation between the two methods is expected.

In summary, the proposed vision-based measurement method offers significant advantages, including non-contact operation, high spatial resolution, and full-field coverage. It also exhibits strong robustness and engineering applicability under actual fatigue loading conditions. Particularly in scenarios where the installation of traditional strain gages is limited, the number of sensors is insufficient, or synchronous multi-point acquisition is difficult to achieve, this method serves as an effective alternative or complementary tool, providing a new technical pathway for dynamic deformation monitoring of large-scale structures such as wind turbine blades.

## 5. Discussion

The proposed method achieves millimeter-level measurement accuracy, with target recognition and center localisation rates exceeding 99%. Mismatches are effectively eliminated using an epipolar constraint-based rejection strategy, ensuring reliable trajectory reconstruction. In addition, the influence of motion blur is negligible due to the low deformation frequency (0.5 Hz) and small inter-frame displacement at 12 fps. Robustness to partial occlusion is ensured through epipolar constraint-based filtering, which effectively removes mismatched and occluded feature points.

In terms of computational efficiency, the processing cost is mainly concentrated in the initial frame, where full template matching and feature extraction are performed. For subsequent frames, the search is restricted to a small local window (10 × 10 pixels) around previously detected positions, and only local feature refinement is carried out. This strategy significantly reduces computational load and ensures that processing speed is only weakly affected by the number of targets and image resolution. As a result, the system can process thousands of frames per minute, demonstrating strong real-time capability.

Experimental results show smooth elliptic breathing deformation near the blade root without outliers. Beyond 10 m, the deformation trajectories remain approximately elliptic but exhibit slight jitter, which is mainly attributed to the short camera baseline. Overall, the method demonstrates good robustness across both single-cycle and multi-cycle deformation measurements, enabling accurate recovery of 3D blade deformation.

From an engineering perspective, a key design consideration lies in the selection of the target type under challenging illumination conditions. Although coded retro-reflective targets offer high automatic recognition rates under coaxial illumination, their performance degrades significantly under strong non-coaxial LED lighting inside the blade, leading to contrast loss and decoding failure. Therefore, high-precision non-reflective targets are adopted in this study. While this choice sacrifices some degree of automation, it ensures stable sub-pixel localisation under complex lighting conditions, representing a practical trade-off for internal measurement environments.

Due to spatial constraints inside the blade, the stereo baseline (0.42–0.48 m) is relatively short. This configuration avoids installation difficulties and field-of-view overlap issues associated with larger baselines. However, from a geometric perspective, the short baseline inevitably increases sensitivity to depth (Z-direction) error, particularly for long-distance targets. To mitigate this effect, template matching combined with epipolar constraints is employed to maintain high recognition accuracy. Nevertheless, a slight degradation in far-field depth accuracy is observed, which is consistent with stereo vision theory. Future improvements could include optimized camera layouts or multi-camera configurations to enhance depth accuracy.

To ensure reliable performance evaluation, accuracy assessment is conducted using not only conventional reprojection errors but also a multi-depth planar target validation method. This approach systematically evaluates the spatial distribution of measurement accuracy across the working volume. The results show that Experiment 1 achieves a mean point position error of 0.03 cm and a plane fitting standard deviation of 0.02 cm, while Experiment 2 yields corresponding values of 0.06 cm and 0.07 cm. These results demonstrate consistent and stable system performance within the effective measurement range, confirming the validity of the calibration strategy.

Compared with alternative vision-based approaches, the proposed method exhibits distinct advantages in constrained internal environments. Target-free methods (e.g., DIC or natural feature tracking) rely heavily on surface texture and stable illumination, making them less reliable in low-texture and uneven lighting conditions inside blades. Monocular 3D methods offer flexible deployment but generally suffer from limited depth accuracy and reduced robustness under occlusion and large deformation. In contrast, the proposed stereo-based approach, combined with artificial targets and tailored calibration, provides more reliable and accurate 3D deformation measurement under confined, low-light, and high H/B ratio conditions.

The method offers several core advantages, including full-field non-contact measurement and high dynamic monitoring capability, enabling real-time capture and visualization of global deformation distribution. Building upon the dedicated measurement strategy for confined internal environments, the system maintains stable performance under restricted space, severe occlusion, and challenging illumination conditions.

By integrating feature-based template matching with subpixel localisation and robust corner extraction, the method ensures reliable target detection and tracking even under long-distance observation and high height-to-baseline (H/B) ratio configurations. In addition, the relative calibration framework enhances geometric consistency between cameras, contributing to improved 3D reconstruction accuracy under constrained setups.

These features collectively enable accurate and continuous recovery of full cross-sectional deformation, achieving millimeter-level dynamic 3D measurement during fatigue testing. As a result, the proposed approach provides an effective and practical tool for dynamic deformation monitoring and performance evaluation of wind turbine blades, particularly in complex internal environments where conventional methods are difficult to apply.

The method’s core advantages include full-field non-contact measurement and high dynamic monitoring capability, enabling real-time capture of global deformation distribution with visualized output, providing an effective tool for dynamic deformation monitoring and performance evaluation of wind turbine blades. Limitations remain: (1) occasional misidentification at >10 m; (2) z-direction error amplification with distance due to baseline constraints; and (3) synchronization errors (~10 ms) from soft triggering. Future work can optimize camera layout, improve synchronization, and enhance calibration to increase long-distance reliability.

## 6. Conclusions

This paper proposes a vision-based measurement system for blade breath deformation, which analyzes the transverse and longitudinal response characteristics of blade deformation by extracting deformation data from targets on different cross-sections. The proposed method offers high accuracy, non-contact operation, and ease of use, making it well-suited for the complex working environment inside the blade and capable of accurately identifying target markers. The system employs a matching approach that combines template- and feature-based methods, significantly enhancing recognition accuracy and demonstrating great potential for application in displacement measurement of large-scale equipment.

Through the deformation test of two sets of blade fatigue experiments, the dynamic deformation measurement method proposed in this paper can accurately capture the complete cycle experimental data of multiple sections during fatigue loading, reconstruct the deformation and displacement data of the fatigue loading cycle, and accurately record the fatigue experiments’ running cycle and the deformation characteristics under the peak value. In addition, the system allows for more accurate calculations of localized focus areas, significantly reducing test costs. The blade-breathing deformation data can be further used to calculate the changes in displacement field, strain field, and strain rate during blade loading; to verify the theory of fatigue research and modeling simulation computational analysis to explore the emergence of cracks and their evolution laws, and to further reveal the intrinsic mechanism of blade failure [32,33,34].

In the experiments of this paper, we use two cameras as stereo cameras to measure the breath deformation inside the blade. Compared with the method of deformation measurement of the blade on the outside, the method of this paper can be more comprehensive in deformation measurements of the whole cross-section of the blade and is less subject to the interference of the external environment on the camera. From the resultant data of the target cross-section, two camera pairs existed with partially missing target fields of view, which affected the monitoring of the complete cross-section. In the future, we plan to increase the number of cameras to ensure complete coverage of the target cross-section by optimizing the spatial layout and baseline length configuration of the cameras, as well as adopting the hardware trigger mode to replace the existing soft trigger scheme to eliminate time synchronization errors and improve the timing consistency of the multi-camera data acquisition.

## Figures and Tables

**Table 1:** List of blade deformation tests.

Serial Number	Typology	Name	Quantities	Clarification
1	Hardware	Industrial camera	2	High-resolution cameras for stereo image acquisition, mounted on stable brackets
2		Magnetic mounting bracket	2	Immobilizes the system on the blade and minimizes transmitted vibrations
3		Gimbal	1	Fixes cameras and allows precise angle adjustment
4		Light source	2	Provides supplemental illumination inside the dark blade cavity
5		Calibration board	1	High-precision alumina checkerboard for camera calibration
6		Non-reflective targets	several	Applied to blade cross-sections for deformation analysis
7	Software	Acquisition System		Custom software for synchronized image capture
8		Data processing system		Vision-based blade breathing deformation monitoring system, developed using OpenCV and custom algorithms

**Table 2:** Experiment 1 error analysis.

Calibration Plates for Different Positional Attitudes	X-Direction Error (cm)	Y-Direction Error (cm)	Z-Direction Error (cm)	Point Error (cm)
1	0.00619329	0.00613667	0.0263174	0.027724
2	0.0194112	0.00736955	0.0261335	0.0333776
3	0.0193307	0.0121259	0.0271333	0.0354532
4	0.00678098	0.00589091	0.0287487	0.0301193
5	0.0201809	0.00651468	0.0278934	0.0350393
6	0.0228586	0.00710186	0.0270391	0.0361118
7	0.00753229	0.0117332	0.0267279	0.030146
8	0.00901402	0.00953557	0.0327516	0.0352824

**Table 3:** Experiment 2 error analysis.

Calibration Plates for Different Positional Attitudes	X-Direction Error (cm)	Y-Direction Error (cm)	Z-Direction Error (cm)	Point Error (cm)
1	0.0124	0.0122	0.0526	0.0554
2	0.0388	0.0148	0.0522	0.0668
3	0.0386	0.0242	0.0542	0.0708
4	0.0136	0.0118	0.0574	0.0602
5	0.0402	0.0130	0.0558	0.0702
6	0.0458	0.0142	0.0540	0.0722
7	0.0150	0.0234	0.0534	0.0602
8	0.0180	0.0190	0.0656	0.0706

**Table 4:** Comparison of peak deformation values measured by vision system and strain gages under fatigue loading.

Target Number	Max Deformation in Experiment 1 (mm)	Max Deformation—Strain Gage (mm)	Deviation	Max Deformation in Experiment 2 (mm)	Max Deformation—Strain Gage (mm)	Deviation (mm)
0	11.2	10.7	0.5	41.6	40.9	0.7
1	16.7	17.2	0.5	48.3	47.6	0.7
2	30.8	29.6	1.2	52.4	51.1	1.3
3	28.7	28.3	0.4	57.9	56.8	1.1
4	31.6	32.5	0.9	62.5	63.4	1.1
5	27.2	26.3	0.9	68.3	67.4	0.9
6	24.6	23.7	1.1	53.6	52.9	0.7
7	21.3	20.6	0.7	62.8	61.6	0.8
8	18.8	18.2	0.6	69.2	68.4	0.8
9	16.9	15.2	1.6	78.8	80.0	1.2
10	12.2	13.1	0.9			
11	10.9	10.1	0.8			

## Data Availability

All data generated or analyzed during this study are included in this article. The datasets are also available from the corresponding authors upon reasonable request.
